# Knowledge, attitudes and practices among patients with periodontal disease toward disease management

**DOI:** 10.3389/fpubh.2024.1500586

**Published:** 2024-12-06

**Authors:** Siyu Zhao, Yanyun Wu

**Affiliations:** ^1^Nanjing Stomatological Hospital, Affiliated Hospital of Medical School, Institute of Stomatology, Nanjing University, Nanjing, China; ^2^Department of Stomatology, Nanjing Drum Tower Hospital, The Affiliated Hospital of Nanjing University Medical School, Nanjing, China

**Keywords:** knowledge, attitudes, practices, cross-sectional study, oral health, periodontal disease

## Abstract

**Introduction:**

Periodontal disease is a common chronic inflammatory condition that affects oral health and carries broader public health implications. This study aimed to assess the levels of knowledge, attitudes and practices (KAP) among patients with periodontal disease toward disease management.

**Methods:**

This cross-sectional web-based study was conducted between March 2022 and March 2023. A self-administered questionnaire was designed to evaluate KAP toward disease management.

**Results:**

A total of 514 questionnaires were collected. Among the patients, 313 (60.89%) of them were female, 309 (60.12%) resided in urban areas and 130 (25.29%) reported having severe periodontal disease. The mean scores of knowledge, attitudes and practices were 3.05 ± 2.03 (possible range: 0–8), 18.71 ± 3.64 (possible range: 6–30), and 14.85 ± 3.63 (possible range: 5–25), respectively. The knowledge item with highest correctness rate was the link between periodontal disease and systemic health (60.70%), while the lowest correctness rate was seen for understanding disease classification (36.96%). Pearson’s analysis revealed positive correlations between knowledge and attitude (*r* = 0.31, *p* < 0.001), knowledge and practices (*r* = 0.23, *p* < 0.001) attitudes, as well as attitudes and practices (*r* = 0.17, *p* < 0.001). Multivariate logistic regression analysis showed that knowledge (OR = 1.30, 95% CI: 1.14–1.49, *p* < 0.001), age ≥ 33 years old (OR = 0.33, 95% CI: 0.18–0.62, *p* = 0.001), housewife/househusband (OR = 0.41, 95% CI: 0.17–0.95, *p* = 0.037) and student (OR = 0.43, 95% CI: 0.20–0.92, *p* = 0.029) were independently associated with practices.

**Conclusion:**

Patients with periodontal disease had insufficient knowledge, negative attitudes, and passive practices toward disease management. Further efforts could be directed toward enhancing patient education on periodontal disease to improve knowledge, thereby positively influencing attitudes and disease management practices.

## Introduction

Periodontal disease, also known as gum disease, is a chronic inflammatory condition that affects the supporting structures of the teeth, including the gums, periodontal ligament, and alveolar bone (1). The impact of periodontal disease extends beyond individual oral health presenting significant implications for public health on a global scale (2). It’s high prevalence and associated health and economic burdens underscore the importance of effective prevention and treatment strategies (3, 4).

In China, a national survey revealed that periodontal disease among adults aged 55–74 was characterized by high prevalence rates of bleeding, probing depths ≥4 mm, and attachment loss >3 mm, underlining the need for targeted public health interventions (5). It is widely recognized that the progression of periodontal disease is influenced by multiple of factors, including microbial pathogens, immune response, tobacco use, and systemic illnesses. In conjunction with the aforementioned biopathological factors, inadequate oral hygiene is a particularly prominent risk element for the initiation of periodontitis. However, it is frequently underappreciated (6, 7). Several studies have demonstrated that periodontal disease patients’ knowledge and attitude toward disease management are potentially important factors affecting their oral health behavior. A cross-sectional study with a sample of 250 adult users of primary health care services in Brazil demonstrated that primary health care users with low oral health literacy exhibited more severe periodontal diseases (8). Similarly, a study on inequities in periodontal disease prevalence, prevention, and management concluded that the frequency of dental visits, oral hygiene, and risk behaviors of patients which impact an individual’s oral health status (9). However, comprehensive study encompassing these facets is conspicuously absent.

Knowledge, attitudes and practices (KAP) studies have been widely used in clinical research (10–12). This provides insights into the determinants that influence decisions and actions, rendering it valuable for crafting precise interventions. Moreover, it contributes to attaining a comprehensive comprehension of the intricate interplay among KAP. Globally, most prior KAP studies on periodontal disease have focused on healthcare professionals (13), pregnant women (14), or other population groups (15), primarily addressing diagnosis, treatment, or prevention, with few studies specifically examining KAP among patients with periodontal disease concerning self-management. Only one study conducted in China reported satisfactory levels of knowledge and attitudes but highlighted gaps in self-management practices related to dental plaque control among these patients (16). Therefore, this study aimed to investigate the KAP among patients with periodontal disease toward disease management.

## Materials and methods

### Study design and participants

This web-based cross-sectional study was conducted between March 2022 and March 2023 at the Department of Dentistry, Nanjing Drum Tower Hospital and the First Outpatient Department of Nanjing Stomatological Hospital among patients with periodontal disease. The inclusion criteria were as follows: (1) aged above 18 years, (2) willing to provide informed consent and participate voluntarily, (3) able to communicate effectively in the local language, and (4) a confirmed diagnosis of periodontal disease (gingivitis or periodontitis) from a qualified dentist. Participants with severe cognitive impairment or mental health conditions affecting communication and comprehension were excluded.

### Questionnaire

The questionnaire was developed following the Consensus of Chinese stomatological multidisciplinary experts on maintaining periodontal health (First edition) and several related studies (17–19), and subjected to scrutiny by three senior dental specialists. A small-scale pre-test was conducted (*n* = 59), with a Cronbach’s *α* value of 0.751, indicating good internal reliability. The questionnaire included cutoffs for knowledge, attitudes, and practices, set at 4, 21, and 17, respectively. These cutoffs correspond to 70% of the total score for each dimension, a criterion chosen to represent sufficient knowledge, positive attitudes, and proactive practices. This threshold is widely used in KAP studies for clear and consistent classification.

The final questionnaire consisted of four dimensions: demographic characteristics, knowledge, attitudes and practices. The selection of demographic characteristics was primarily based on clinical experience, expert consultation, and findings from previous studies. For instance, we included basic demographic characteristics (e.g., gender, age, place of residence), as well as variables related to periodontal disease that are easily self-reported, such as disease type, severity, smoking and drinking habits, and brushing frequency. To enhance response accuracy and quality control, two “trap questions” (K3 and K10) were incorporated into the knowledge dimension. The concept of trap questions involves presenting conflicting statements on the same topic to detect inconsistencies in responses. In this study, K3 stated that “periodontal disease is closely associated with systemic diseases,” whereas K10 stated that it is “completely independent of systemic diseases.” If respondents provided the same response to both questions, the questionnaire was considered logically inconsistent and excluded from analysis. Thus, the knowledge dimension consists of 10 questions, with 1 point for the correct answer and 0 points for the rest, resulting in a total score range of 0–8 after excluding trap questions. The attitude section consists of 6 questions, using a 5-point Likert scale, with each question being assigned a score of 1 point to 5 points for the options from very positive to very negative, with a possible range of 6–30 points. The practice section consists of 5 questions, also using a 5-point Likert scale, with each question being assigned a score of 1 point to 5 points from the highest to the lowest frequency of the practice, with a possible range of 5–25 points.

The data were collected using an online questionnaire hosted on Sojump platform^1^. The online questionnaire was distributed through WeChat, Internet forums, and web links. All data were collected anonymously. To prevent duplication, an IP restriction was implemented, ensuring that each survey could only be completed once per unique IP address.

### Statistical analysis

STATA 17.0 (Stata Corporation, College Station, TX, United States) was used for statistical analysis. The continuous variables were expressed as mean ± SD, and the categorical variables were expressed as *n* (%). The continuous variables that conformed to a normal distribution were tested by the *t*-test or ANOVA. Pearson correlation was used to analyze the correlation between knowledge, attitudes, and practices. For multivariate analysis, the cutoff scores for knowledge, attitudes, and practices were established as 4, 21, and 17, respectively, based on 70% of the maximum possible score for each section. This method was selected to ensure that the classification accurately reflects sufficient levels in each category, as used in previous KAP research (20). Scores surpassing this threshold were categorized as indicative of sufficient knowledge, positive attitudes, and proactive practices. As a result, the designated cutoffs for knowledge, attitudes, and practices were set at 4, 21, and 17, respectively. Scores surpassing this threshold were categorized as indicative of sufficient knowledge, positive attitudes, and proactive practices. Two-sided *p* < 0.05 was considered statistically significant in this study.

## Results

A total of 514 questionnaires were collected. Among the patients, 313 (60.89%) were female, 309 (60.12%) resided in urban and 130 (25.29%) reported having severe periodontal disease. The mean score of knowledge, attitudes and practices were 3.05 ± 2.03 (possible range: 0–8), 18.71 ± 3.64 (possible range: 6–30), and 14.85 ± 3.63 (possible range: 6–30), respectively (Table 1).

**Table 1:** Knowledge, attitude, and practice scores and demographic characteristics.

Variables	*N* (%)	Knowledge		Attitudes		Practice score	
		Mean ± SD	*P*	Mean ± SD	*P*	Mean ± SD	*P*
**Total**	514	3.05 ± 2.03		18.71 ± 3.64		14.85 ± 3.63	
**Gender**			0.006		0.102		0.504
Male	201 (39.11)	2.75 ± 1.72		18.38 ± 3.38		14.72 ± 3.68	
Female	313 (60.89)	3.25 ± 2.19		18.92 ± 3.79		14.94 ± 3.60	
**Age, years**			0.617		0.142		<0.001
≤22	134 (26.07)	3.01 ± 1.87		18.93 ± 3.66		15.40 ± 3.47	
23–27	128 (24.90)	3.14 ± 1.99		18.10 ± 3.50		15.01 ± 3.18	
28–32	124 (24.12)	3.19 ± 2.11		19.10 ± 3.60		15.39 ± 3.93	
≥33	128 (24.90)	2.88 ± 2.16		18.70 ± 3.76		13.60 ± 3.66	
**Residence**			<0.001		0.028		0.171
Rural	205 (39.88)	2.53 ± 1.58		18.27 ± 3.68		14.58 ± 3.35	
Urban	309 (60.12)	3.40 ± 2.22		18.99 ± 3.59		15.03 ± 3.80	
**Education**			<0.001		0.055		0.143
Middle school or below	84 (16.34)	2.49 ± 1.27		18.33 ± 3.67		14.50 ± 3.88	
High school and Technical secondary school	116 (22.57)	2.51 ± 1.58		18.04 ± 3.40		14.35 ± 3.30	
Junior college and Undergraduate	174 (33.85)	3.68 ± 2.28		19.09 ± 33.48		15.28 ± 3.55	
Postgraduate or above	140 (27.24)	3.07 ± 2.19		19.00 ± 3.94		14.94 ± 3.82	
**Occupation**			<0.001		<0.001		<0.001
Employee	102 (19.84)	4.53 ± 2.54		20.40 ± 3.61		16.49 ± 3.79	
Part-time	53 (10.31)	2.49 ± 1.37		17.94 ± 3.00		15.06 ± 3.84	
Freelance	50 (9.73)	2.66 ± 1.78		18.96 ± 3.15		14.22 ± 3.56	
Unemployed	68 (13.24)	2.44 ± 1.19		17.47 ± 3.06		14.35 ± 3.48	
Housewife/househusband	55 (10.70)	2.49 ± 1.41		17.75 ± 3.51		14.11 ± 3.13	
Student	69 (13.42)	3.42 ± 2.53		18.65 ± 3.67		14.42 ± 3.42	
Retired	54 (10.51)	2.93 ± 1.61		18.72 ± 3.75		14.70 ± 4.11	
Other	63 (12.26)	2.32 ± 1.27		18.62 ± 4.19		14.30 ± 2.92	
Medical insurance (multiple choices)							
No medical insurance	241 (46.89)						
Social medical insurance	324 (63.04)						
Social medical insurance and other commercial medical insurance	287 (55.84)						
**Per capita monthly income, CNY**			0.094		0.414		0.421
<2,000	86 (16.73)	2.83 ± 1.74		18.35 ± 3.36		14.85 ± 3.59	
2,000-5,000	94 (18.29)	3.10 ± 2.01		18.87 ± 3.38		15.29 ± 3.60	
5,000-10,000	113 (21.98)	3.16 ± 2.24		18.66 ± 3.41		15.12 ± 3.54	
10,000-20,000	122 (23.74)	3.39 ± 2.23		19.16 ± 3.63		14.63 ± 3.46	
>20,000	99 (19.26)	2.69 ± 1.71		18.34 ± 4.33		14.40 ± 4.00	
**Periodontal disease type**			0.108		0.440		0.067
Unclear	2(0.39)	6.00		22.00		19.00	
Gingivitis	238 (46.30)	3.09 ± 2.03		18.69 ± 3.51		15.12 ± 3.59	
Periodontitis	274 (53.31)	3.00 ± 2.03		18.70 ± 3.76		14.58 ± 3.66	
**Severity of periodontal disease**			<0.001		0.006		0.054
Mild	149 (28.99)	3.77 ± 2.41		19.18 ± 3.64		15.18 ± 3.97	
Moderate	121 (23.54)	3.33 ± 2.09		18.90 ± 3.59		15.36 ± 3.70	
Severe	130 (25.29)	2.30 ± 1.24		17.75 ± 3.65		14.28 ± 3.46	
Unclear	114 (22.18)	2.69 ± 1.79		18.97 ± 3.54		14.53 ± 3.19	
**Smoking habit**			<0.001		<0.001		0.340
No smoking habit	199 (38.72)	4.13 ± 2.46		19.87 ± 3.69		15.15 ± 3.98	
Less than 10 cigarettes per day	164 (31.91)	2.33 ± 1.35		17.84 ± 3.29		14.64 ± 3.38	
More than or equal to 10 cigarettes per day	151 (29.38)	2.42 ± 1.27		18.11 ± 3.55		14.69 ± 3.40	
**Alcohol consumption**			<0.001		0.170		0.904
Yes	206 (40.08)	2.61 ± 1.75		18.44 ± 3.51		14.87 ± 3.47	
No	308 (59.92)	3.35 ± 2.15		18.89 ± 3.72		14.83 ± 3.74	
**Frequency of teeth brushing**			<0.001		0.079		0.110
Once	101 (19.65)	3.21 ± 2.04		18.50 ± 3.40		15.01 ± 3.57	
Twice	167 (32.49)	3.88 ± 2.40		19.29 ± 3.83		15.26 ± 3.66	
3 times	106 (20.62)	2.54 ± 1.73		18.53 ± 3.89		14.84 ± 3.88	
4 times and above	140 (27.24)	2.35 ± 1.25		18.29 ± 3.31		14.26 ± 3.41	
**Dentition defect**			0.558		0.575		0.681
Yes	228 (44.36)	3.00 ± 1.86		18.61 ± 3.38		14.78 ± 3.67	
No	286 (55.64)	3.10 ± 2.16		18.79 ± 3.84		14.91 ± 3.61	
Comorbidities (multiple choices)							
Diabetes mellitus	108 (21.01)						
Osteoporosis	102 (19.84)						
Blood system disorders	100 (19.46)						
Cardiovascular disorders	92 (17.90)						
Immune system disorders	102 (19.84)						
Other	89 (17.32)						
None of the above	167 (32.49)						
Unclear	117 (22.76)						
Oral parafunctional disorders (multiple choices)							
Overbite	208 (40.27)						
Nocturnal teeth grinding	218 (42.41)						
Neither	228 (44.36)						
Unclear	204 (39.44)						
**Radiotherapy or chemotherapy for cancer**			<0.001		0.013		0.141
Yes	179 (34.82)	2.53 ± 1.34		18.16 ± 3.41		15.17 ± 3.28	
No	335 (65.18)	3.34 ± 2.27		19.00 ± 3.73		14.68 ± 3.80	
**Family history of periodontal disease**			0.106		0.741		0.039
Yes	253 (49.22)	3.20 ± 2.10		18.65 ± 3.64		15.19 ± 3.48	
No	261 (50.78)	2.91 ± 1.96		18.76 ± 3.65		14.52 ± 3.76	

The three knowledge items with the highest correctness rates were as follows: “Periodontal disease is closely associated with systemic diseases such as diabetes mellitus and cardiovascular disorders” (K3) with a correctness rate of 60.70%, and “Daily oral hygiene maintenance is an important way to maintain periodontal health” (K7), with a correctness rate of 40.66%. On the other hand, the three items with the lowest correctness rates were “Periodontal disease is divided into two main categories: gingivitis and periodontitis” (K1) with a correctness rate of 36.96%, “Regular oral check-ups with a dental specialist and appropriate therapy are necessary to maintain oral and periodontal health” (K8) with a correctness rate of 37.16%, and “Periodontal disease is a mild disease that can be treated with medication regardless of its severity” (K9) with a correctness rate of 37.74% (Table 2).

**Table 2:** Knowledge.

Knowledge	Correctness *N* (%)
K1. Periodontal disease is divided into two main categories: gingivitis and periodontitis.	190 (36.96)
K2. Periodontal disease does not affect the pronunciation and appearance of patient.	196 (38.13)
K3. Periodontal disease is closely associated with systemic diseases such as diabetes mellitus and cardiovascular disorders.	312 (60.70)
K4. Microbial infections may be a cause of periodontal disease.	199 (38.72)
K5. Periodontal disease is one of the main causes of tooth loosening and loss.	194 (37.74)
K6. Bleeding gums and swollen gums are common symptoms of periodontal disease.	197 (38.33)
K7. Daily oral hygiene maintenance (brushing in the correct manner, using of Interdental cleaning measures such as flossing) is an important way to maintain periodontal health.	209 (40.66)
K8. Regular (half-yearly to yearly) oral check-ups with a dental specialist and appropriate therapy (scaling, scraping, etc.) are the measures necessary to maintain oral and periodontal health.	191 (37.16)
K9. Periodontal disease is a mild disease that can be treated with medication regardless of its severity.	194 (37.74)
K10. Periodontal disease is completely independent with systemic diseases such as diabetes mellitus and cardiovascular disorders.	312 (60.70)

In this study, 46.11% of the participants strongly agreed/agreed that paying close attention to bleeding and painful gums is important in their daily lives (A2). Similarly, 43.78% strongly agreed/agreed that following doctor’s instructions and regular follow-up appointments was essential for achieving a good therapeutic effect (A6). Moreover, a considerable portion (12.65%) of patients indicated a preference for self-managed measures over seeking medical help (A5) (Table 3).

**Table 3:** Attitudes.

	Strongly agree *N* (%)	Agree *N* (%)	Neutral *N* (%)	Disagree *N* (%)	Strongly disagree *N* (%)
A1. You think it is important for patients to stay attentive to periodontal disease management and appropriately learning about relevant knowledge.	120 (23.35)	120 (23.35)	91 (17.70)	86 (16.73)	97 (18.87)
A2. You will pay close attention to the bleeding and painful gums in your daily life.	107 (20.82)	130 (25.29)	100 (19.46)	88 (17.12)	89 (17.32)
A3. You think that periodontal disease is a very common disease and does not usually cause serious hazards.	79 (15.37)	104 (20.23)	106 (20.62)	119 (23.15)	106 (20.62)
A4. The physical and psychological discomfort caused by periodontal disease makes you feel irritable.	97 (18.87)	118 (22.96)	106 (20.62)	86 (16.73)	107 (20.82)
A5. You generally prefer to take your own measures such as taking medication to manage your illness rather than seeking medical help.	65 (12.65)	107 (20.82)	112 (21.79)	126 (24.51)	104 (20.23)
A6. You think it is important to follow your doctor’s instructions and to have regular follow-up appointments for a good therapeutic effect.	103 (20.04)	122 (23.74)	108 (21.01)	85 (16.54)	96 (18.68)

Regarding practices, 18.29% of patients indicated undergoing annual visits to dental clinics or hospital dentistry for oral examinations, while 24.51% exhibited a more rigorous approach with biannual or more frequent visits (P1). Concerning basic periodontal scaling, 21.21% of respondents frequently sought dental intervention, 19.07% adhered to at least an annual schedule, while 16.93% extended their visit gap beyond 5 years (P2). Additionally, a notable trend encompassed the acquisition of knowledge on periodontal disease prevention and treatment, with 22.57% frequently seeking such information and 15.18% consistently engaging in this pursuit (P3). Notably, 25.10% of patients intermittently prioritized the observation of bleeding or swollen gums as a pivotal facet of their oral care regimen (P4) (Table 4).

**Table 4:** Practices.

	*N* (%)				
	Twice and more times per year	Once per year	Once every 2–5 years	Last time more than 5 years ago	No discomfort no oral examination
P1. How often do you visit a dental clinic or hospital dentistry for an oral examination.	94 (18.29)	126 (24.51)	104 (20.23)	84 (16.34)	106 (20.62)
	At least once per year	Once every 2–3 years	Once every 4–5 years	Last time more than 5 years ago	Never
P2. How often you visit a dentist for basic periodontal scaling (dental cleaning).	98 (19.07)	109 (21.21)	93 (10.09)	87 (16.93)	127 (24.71)
	Always	Often	Sometimes	Occasionally	Never
P3. How often do you actively obtain information about prevention and treatment of periodontal disease through various sources (e.g., by participating in in-hospital education, by television and internet, or by consulting your attending physician).	78 (15.18)	116 (22.57)	103 (20.04)	123 (23.93)	94 (18.29)
P4. How often do you pay attention to whether you have bleeding or swollen gums.	79 (15.37)	100 (19.46)	129 (25.10)	100 (19.46)	106 (20.62)
	Very conforming	Conforming	Not necessarily	Not conforming	Very not conforming
P5. You will assess any improvement or worsening of your disease over time and discuss this with your attending physician.	97 (18.87)	119 (23.15)	116 (22.57)	85 (16.54)	97 (18.87)

Pearson’s analysis showed that knowledge and attitudes (*r* = 0.312, *p* < 0.001) were positively correlated as well as knowledge and practices (*r* = 0.230, *p* < 0.001). Additionally, there was a positive correlation between attitudes and practices (*r* = 0.171, *p* < 0.001) (Table 5).

**Table 5:** Pearson’s analysis.

	Knowledge	Attitudes	Practices
Knowledge	1		
Attitudes	0.31 (*P* < 0.001)	1	
Practices	0.23 (*P* < 0.001)	0.17 (*P* < 0.001)	1

The multivariate logistic regression analysis showed that urban residence (OR = 1.84, 95% CI: 1.11–3.05, *p* = 0.018), freelance employment (OR = 0.34, 95% CI: 0.14–0.84, *p* = 0.020), unemployed (OR = 0.25, 95% CI: 0.10–0.63, *p* = 0.003), severe periodontal disease (OR = 0.27, 95% CI: 0.14–0.55, *p* < 0.001) and smoking <10 cigarettes per day (OR = 0.25, 95% CI: 0.14–0.45, *p* < 0.001), smoking ≥10 cigarettes per day (OR = 0.32, 95% CI: 0.17–0.57, *p* < 0.001), alcohol consumption (OR = 0.51, 95% CI: 0.31–0.84, *p* = 0.008), brushing teeth 3 times a day (OR = 0.41, 95% CI: 0.19–0.89, *p* = 0.022) were independently associated with knowledge (Table 6).

**Table 6:** Multivariate analysis of knowledge.

Variables	Univariate		Multivariate	
	OR (95%CI)	*P*	OR (95%CI)	*P*
Gender				
Male	Ref.		Ref.	
Female	1.775 (1.189 2.650)	0.005	1.504 (0.917 2.466)	0.106
Age				
≤22 years old	Ref.		Ref.	
23–27 years old	1.527 (0.903 2.583)	0.114	1.691 (0.879 3.255)	0.116
28–32 years old	1.345 (0.788 2.295)	0.278	1.208 (0.607 2.405)	0.590
≥33 years old	0.985 (0.570 1.702)	0.956	0.802 (0.396 1.625)	0.540
Residence				
Rural	Ref.		Ref.	
Urban	2.203 (1.465 3.312)	<0.001	1.839 (1.109 3.048)	0.018
Education				
Middle school and below	Ref.		Ref.	
High school and Technical secondary school	1.112 (0.566 2.187)	0.758	1.372 (0.640 2.944)	0.416
Junior college and Undergraduate	2.528 (1.384 4.617)	0.003	1.322 (0.644 2.712)	0.447
Postgraduate and above	1.519 (0.804 2.867)	0.198	1.042 (0.488 2.224)	0.915
Occupation				
Employee	Ref.		Ref.	
Part-time	0.222 (0.105 0.472)	<0.001	0.451 (0.181 1.122)	0.087
Freelance	0.240 (0.112 0.511)	<0.001	0.344 (0.141 0.844)	0.020
Unemployed	0.131 (0.060 0.285)	<0.001	0.253 (0.103 0.626)	0.003
Housewife/Househusband	0.259 (0.126 0.533)	<0.001	0.660 (0.277 1.569)	0.347
Student	0.459 (0.246 0.857)	0.015	0.573 (0.262 1.253)	0.163
Retired	0.319 (0.158 0.645)	0.001	0.651 (0.273 1.552)	0.332
Other	0.126 (0.056 0.283)	<0.001	0.181 (0.069 0.477)	0.001
Per capita monthly income, CNY				
<2,000	Ref.		Ref.	
2,000–5,000	1.162 (0.606 2.227)	0.651	0.483 (0.212 1.100)	0.083
5,000–10,000	1.281 (0.689 2.381)	0.434	0.629 (0.288 1.372)	0.244
10,000–20,000	1.601 (0.876 2.925)	0.126	1.171 (0.556 2.463)	0.678
>20,000	0.925 (0.479 1.788)	0.817	0.907 (0.407 2.024)	0.812
Specific types of periodontal disease				
Gingivitis	Ref.		Ref.	
Periodontitis	1.079 (0.739 1.576)	0.692	1.323 (0.813 2.153)	0.259
Severity of periodontal disease				
Mild	Ref.		Ref.	
Moderate	0.799 (0.490 1.301)	0.367	0.947 (0.518 1.731)	0.860
Severe	0.215 (0.120 0.386)	<0.001	0.274 (0.137 0.547)	<0.001
Unclear	0.353 (0.204 0.612)	<0.001	0.514 (0.266 0.996)	0.049
Smoking				
No smoking habit	Ref.		Ref.	
Less than 10 cigarettes/day	0.187 (0.114 0.308)	<0.001	0.247 (0.136 0.449)	<0.001
More than or equal to 10 cigarettes/day	0.216 (0.132 0.355)	<0.001	0.315 (0.173 0.572)	<0.001
Alcohol consumption				
Yes	0.510 (0.341 0.763)	0.001	0.507 (0.308 0.836)	0.008
No	Ref.		Ref.	
Frequency of teeth brushing				
Once	Ref.		Ref.	
Twice	1.693 (1.016 2.819)	0.043	1.629 (0.849 3.126)	0.142
3 times	0.439 (0.232 0.828)	0.011	0.408 (0.189 0.880)	0.022
4 times and above	0.371 (0.202 0.680)	0.001	0.505 (0.245 1.042)	0.064
Dentition defect				
Yes	0.976 (0.669 1.425)	0.902	1.260 (0.782 2.031)	0.343
No	Ref.		Ref.	
Radiotherapy or chemotherapy for cancer				
Yes	0.536 (0.353 0.814)	0.003	0.949 (0.565 1.595)	0.844
No	Ref.		Ref.	
Family history of periodontal disease				
Yes	1.280 (0.879 1.865)	0.198	1.072 (0.664 1.732)	0.776
No	Ref.		Ref.	

Knowledge (OR = 1.16, 95% CI: 1.02–1.32, *p* = 0.021), unemployed (OR = 0.28, 95% CI: 0.12–0.65, *p* = 0.003), housewife/househusband (OR = 0.40, 95% CI: 0.17–0.93, *p* = 0.033), student (OR = 0.47, 95% CI: 0.30–0.97, *p* = 0.040) and smoking <10 cigarettes per day (OR = 0.50, 95% CI: 0.29–0.86, *p* = 0.012) and smoking ≥10 cigarettes per day (OR = 0.57, 95% CI: 0.33–0.98, *p* = 0.041) were independently associated with attitudes (Table 7).

**Table 7:** Multivariate analysis of attitudes.

Variables	Univariate		Multivariate	
	OR (95%CI)	*P*	OR (95%CI)	*P*
Knowledge	1.318 (1.200 1.447)	<0.001	1.160 (1.023 1.316)	0.021
Gender				
Male	Ref.		Ref.	
Female	1.275 (0.867 1.876)	0.217	1.000 (0.650 1.540)	0.999
Age				
≤22 years old	Ref.		Ref.	
23–27 years old	0.638 (0.373 1.090)	0.100	0.567 (0.314 1.025)	0.060
28–32 years old	0.814 (0.482 1.375)	0.441	0.661 (0.360 1.213)	0.182
≥33 years old	1.110 (0.669 1.842)	0.686	1.118 (0.625 2.001)	0.707
Residence				
Rural	Ref.		Ref.	
Urban	1.286 (0.875 1.889)	0.200	0.878 (0.563 1.371)	0.567
Education				
Middle school and below	Ref.		Ref.	
High school and Technical secondary school	0.722 (0.379 1.375)	0.322	0.685 (0.344 1.367)	0.283
Junior college and Undergraduate	1.491 (0.848 2.621)	0.165	0.961 (0.511 1.807)	0.901
Postgraduate and above	1.263 (0.701 2.277)	0.437	0.998 (0.525 1.900)	0.996
Occupation				
Employee	Ref.		Ref.	
Part-time	0.293 (0.138 0.620)	0.001	0.442 (0.191 1.025)	0.057
Freelance	0.389 (0.188 0.806)	0.011	0.498 (0.218 1.134)	0.097
Unemployed	0.172 (0.079 0.374)	<0.001	0.279 (0.120 0.652)	0.003
Housewife/Househusband	0.279 (0.132 0.590)	0.001	0.399 (0.172 0.929)	0.033
Student	0.468 (0.247 0.886)	0.020	0.471 (0.304 0.965)	0.040
Retired	0.543 (0.275 1.072)	0.078	0.666 (0.304 1.459)	0.310
Other	0.537 (0.281 1.025)	0.059	0.791 (0.373 1.680)	0.542
*Per capita* monthly income, CNY				
<2,000	Ref.		Ref.	
2,000-5,000	1.096 (0.574 2.091)	0.781	0.824 (0.400 1.696)	0.599
5,000-10,000	1.159 (0.625 2.149)	0.639	0.922 (0.461 1.842)	0.818
10,000-20,000	1.619 (0.892 2.937)	0.113	1.419 (0.724 2.781)	0.308
>20,000	1.019 (0.536 1.938)	0.955	1.016 (0.504 2.051)	0.964
Specific types of periodontal disease				
Gingivitis	Ref.		Ref.	
Periodontitis	0.909 (0.625 1.321)	0.616	0.880 (0.577 1.341)	0.552
Severity of periodontal disease				
Mild	Ref.		Ref.	
Moderate	0.829 (0.498 1.381)	0.472	1.080 (0.612 1.906)	0.791
Severe	0.543 (0.320 0.922)	0.024	0.888 (0.482 1.637)	0.704
Unclear	1.017 (0.612 1.692)	0.947	1.508 (0.840 2.706)	0.168
Smoking				
No smoking habit	Ref.		Ref.	
Less than 10 cigarettes/day	0.341 (0.214 0.541)	<0.001	0.499 (0.290 0.856)	0.012
More than or equal to 10 cigarettes/day	0.379 (0.238 0.605)	<0.001	0.566 (0.328 0.976)	0.041
Alcohol consumption				
Yes	0.711 (0.483 1.047)	0.084	0.910 (0.586 1.413)	0.674
No	Ref.		Ref.	
Frequency of teeth brushing				
Once	Ref.		Ref.	
Twice	1.374 (0.815 2.316)	0.233	1.049 (0.577 1.908)	0.874
3 times	0.851 (0.469 1.544)	0.596	1.038 (0.532 2.026)	0.913
4 times and above	0.719 (0.407 1.268)	0.254	0.925 (0.489 1.751)	0.811
Dentition defect				
Yes	0.934 (0.642 1.360)	0.722	1.016 (0.669 1.545)	0.939
No	Ref.		Ref.	
Radiotherapy or chemotherapy for cancer				
Yes	0.653 (0.436 0.978)	0.039	0.890 (0.560 1.414)	0.620
No	Ref.		Ref.	
Family history of periodontal disease				
Yes	1.047 (0.721 1.518)	0.811	1.050 (0.689 1.601)	0.819
No	Ref.		Ref.	

Knowledge (OR = 1.30, 95% CI: 1.14–1.49, *p* < 0.001), aged ≥33 years old (OR = 0.33, 95% CI: 0.18–0.62, *p* = 0.001), housewife/househusband (OR = 0.41, 95% CI: 0.17–0.95, *p* = 0.037) and student (OR = 0.43, 95% CI: 0.20–0.92, *p* = 0.029) were independently associated with practices (Table 8).

**Table 8:** Multivariate analysis of practices.

Variables	Univariate		Multivariate	
	OR (95%CI)	*P*	OR (95%CI)	*P*
Knowledge	1.311 (1.194 1.439)	<0.001	1.302 (1.141 1.485)	<0.001
Attitudes	1.085 (1.030 1.134)	<0.001	1.037 (0.975 1.103)	0.242
Gender				
Male	Ref.		Ref.	
Female	1.122 (0.768 1.639)	0.553	1.044 (0.679 1.607)	0.844
Age				
≤22 years old	Ref.		Ref.	
23–27 years old	0.749 (0.453 1.240)	0.262	0.641 (0.364 1.127)	0.123
28–32 years old	0.904 (0.548 1.492)	0.694	0.732 (0.412 1.299)	0.286
≥33 years old	0.360 (0.206 0.628)	<0.001	0.333 (0.177 0.628)	0.001
Residence				
Rural	Ref.		Ref.	
Urban	1.368 (0.934 2.003)	0.108	1.147 (0.740 1.779)	0.540
Education				
Middle school and below	Ref.		Ref.	
High school and Technical secondary school	0.850 (0.459 1.574)	0.605	0.849 (0.434 1.659)	0.632
Junior college and Undergraduate	1.397 (0.802 2.431)	0.237	1.065 (0.565 2.007)	0.845
Postgraduate and above	1.022 (0.570 1.834)	0.941	0.874 (0.457 1.672)	0.685
Occupation				
Employee	Ref.		Ref.	
Part-time	0.606 (0.308 1.193)	0.147	0.794 (0.359 1.756)	0.568
Freelance	0.429 (0.209 0.879)	0.021	0.620 (0.276 1.393)	0.247
Unemployed	0.333 (0.170 0.653)	0.001	0.463 (0.213 1.006)	0.052
Housewife/Househusband	0.279 (0.132 0.590)	0.001	0.405 (0.173 0.948)	0.037
Student	0.437 (0.230 0.832)	0.012	0.430 (0.202 0.916)	0.029
Retired	0.421 (0.209 0.849)	0.016	0.505 (0.224 1.141)	0.100
Other	0.370 (0.188 0.728)	0.004	0.570 (0.258 1.257)	0.164
*Per capita* monthly income, CNY				
<2,000	Ref.		Ref.	
2,000–5,000	1.584 (0.850 2.952)	0.148	1.694 (0.835 1.435)	0.144
5,000–10,000	1.389 (0.760 2.539)	0.285	1.256 (0.636 2.482)	0.512
10,000–20,000	0.982 (0.534 1.804)	0.952	0.769 (0.382 1.548)	0.461
>20,000	1.112 (0.592 2.089)	0.740	1.405 (0.695 2.840)	0.344
Specific types of periodontal disease				
Gingivitis	Ref.		Ref.	
Periodontitis	0.691 (0.477 1.001)	0.050	0.769 (0.508 1.165)	0.215
Severity of periodontal disease				
Mild	Ref.		Ref.	
Moderate	1.365 (0.835 2.233)	0.215	1.485 (0.857 2.573)	0.159
Severe	0.591 (0.351 0.996)	0.048	0.924 (0.506 1.686)	0.797
Unclear	0.707 (0.417 1.199)	0.198	0.876 (0.479 1.599)	0.665
Smoking				
No smoking habit	Ref.		Ref.	
Less than 10 cigarettes/day	0.664 (0.426 1.035)	0.071	1.381 (0.791 2.413)	0.257
More than or equal to 10 cigarettes/day	0.747 (0.477 1.170)	0.202	1.707 (0.976 2.986)	0.061
Alcohol consumption				
Yes	0.973 (0.668 1.418)	0.889	1.167 (0.754 1.807)	0.489
No	Ref.		Ref.	
Frequency of teeth brushing				
Once	Ref.		Ref.	
Twice	0.948 (0.571 1.572)	0.835	0.881 (0.492 1.575)	0.668
3 times	0.602 (0.337 1.076)	0.087	0.778 (0.405 1.495)	0.451
4 times and above	0.508 (0.293 0.883)	0.016	0.767 (0.413 1.425)	0.401
Dentition defect				
Yes	0.837 (0.577 1.214)	0.348	0.792 (0.521 1.204)	0.275
No	Ref.		Ref.	
Radiotherapy or chemotherapy for cancer				
Yes	1.219 (0.831 1.788)	0.311	1.516 (0.963 2.388)	0.073
No	Ref.		Ref.	
Family history of periodontal disease				
Yes	1.066 (0.738 1.541)	0.733	0.905 (0.596 1.374)	0.639
No	Ref.		Ref.	

## Discussion

In this study, patients with periodontal disease demonstrated insufficient knowledge, negative attitudes, and passive practices. Suggested recommendations to improve clinical practice include in-hospital training, tailored communication, targeted interventions for specific groups, smoking cessation counseling, and public health campaigns.

The participants demonstrated relatively good understanding of the association between periodontal disease and systemic conditions such as diabetes mellitus and cardiovascular disorders. However, this study identified conspicuous gaps in understanding regarding the consequences of periodontal disease on speech articulation and facial appearance, along with the effectiveness of medical interventions, regardless of the severity of the disease. Beyond their role in the masticatory process, teeth substantially contribute to the articulation of sounds, aesthetic presentation, and the growth of the craniofacial complex (21). A targeted educational approach that addresses these relatively underexplored dimensions has the potential to enhance overall levels of awareness, thus fostering improved outcomes within the domain of oral health (22). Moreover, demographic differences in KAP outcomes were observed, with higher education levels and urban residents showing better knowledge and more proactive practices. Additionally, female patients generally demonstrated higher knowledge scores compared to males, aligning with previous findings that gender differences may influence health-related knowledge acquisition (23). These differences highlight the need for tailored educational interventions that consider socioeconomic and demographic characteristics.

While a significant portion of the patients exhibited an inclination toward highlighting the significance of adhering to medical directives and participating in scheduled post-treatment sessions, certain misconceptions endured among a subset of participants who regarded periodontal disease with reduced gravity. Dispelling these misconceptions and educating patients about the potential consequences of untreated periodontal disease can foster more informed and proactive attitudes toward oral health (24). To address the misconceptions about the severity of periodontal disease, a more nuanced educational strategy is needed. This could involve tailored communication campaigns that emphasize the potential long-term consequences of untreated periodontal disease, such as bone loss, systemic health risks, and impacts on overall well-being. In addition, implementing in-hospital training modules that use real-life cases, visual aids, and interactive workshops could help bridge these knowledge gaps. Providing targeted education to high-risk groups, integrating oral health information into community outreach programs, and utilizing digital tools like mobile health apps may also prove effective in enhancing awareness and promoting timely treatment (25, 26). Dental practitioners ought to actively participate in patient-centered dialogs, aimed at dispelling misconceptions and underscoring the gravity of periodontal ailment. Motivating patients to adhere to dentists’ directives and to consistently attend scheduled follow-up consultations holds the potential to bolster constructive dispositions regarding both preventive measures and therapeutic interventions (27, 28).

Regarding practices, although the majority of participants reported adhering to routine dental visits, a substantial proportion acknowledged never having undergone fundamental periodontal scaling procedures. Additionally, differences were observed in the inclination to seek information concerning periodontal disease, and discrepancies emerged in the frequency of self-monitoring practices in this study. Encouragement of regular dental check-ups and consistent self-monitoring regimens can significantly aid in the early identification of periodontal anomalies and the cultivation of proactive oral health behaviors (29, 30). Dental practitioners assume a pivotal role in educating patients about effective self-monitoring strategies for gum health, encompassing regular assessments for indicators such as gum bleeding or inflammation (31, 32). This emphasis on self-awareness and prompt identification of oral health issues empowers patients to adopt a proactive stance toward their oral care, thereby contributing to enhanced oral health outcomes and holistic well-being.

It is imperative to acknowledge the variability in dental attendance frequency exhibited among the participants, wherein a notable proportion reported having their last visit more than 5 years ago. To enhance the rates of dental attendance, the implementation of diverse strategies emerges as a pivotal consideration. Reminiscent of effective tactics encompassing reminder systems, outreach initiatives, and community-oriented oral health endeavors, these approaches can play a pivotal role in bolstering early detection and intervention for cases of periodontal disease (33–35).

This study had limitations. Primarily, it was conducted at a single center and followed an observational approach. Moreover, it is crucial to acknowledge that the utilization of self-reported data concerning attitudes and practices might render it susceptible to diverse biases, encompassing the likes of social desirability bias or recall bias (36). These biases could potentially lead to overreporting of positive behaviors or underreporting of negative ones, thus affecting the accuracy of the findings. Additionally, the study did not account for all possible factors that might influence KAP, which may limit the depth of the analysis. This limitation has been acknowledged as it is impossible to collect all relevant variables. Future research could mitigate these biases by incorporating objective measures, such as clinical assessments or validated behavior tracking tools, to complement self-reported data. This study presents noteworthy strengths and holds significant clinical implications. In a clinical context, understanding the knowledge gaps, attitudes, and practices of patients with periodontal disease has direct relevance for healthcare practitioners.

## Conclusion

In conclusion, patients with periodontal disease showed insufficient knowledge, negative attitudes, and passive practices toward disease management. Based on the results, clinical practice can be improved by implementing in-hospital training programs to educate patients on periodontal disease prevention and treatment. Targeted interventions should focus on improving practices among specific groups, and smoking cessation counseling should be integrated into patient care protocols.

## Data Availability

The original contributions presented in the study are included in the article/supplementary material, further inquiries can be directed to the corresponding author.

## Footnotes

^1^http://www.sojump.com
